# Proteases in Malaria Parasites - A Phylogenomic Perspective 

**DOI:** 10.2174/138920211797248565

**Published:** 2011-09

**Authors:** Hong Cai, Rui Kuang, Jianying Gu, Yufeng Wang

**Affiliations:** 1 Department of Biology, University of Texas at San Antonio, San Antonio, TX 78249, USA; 2 Department of Computer Science and Engineering, University of Minnesota, Twin Cities, Minneapolis, MN 55455, USA; 3 Department of Biology, College of Staten Island, City University of New York, Staten Island, NY 10314, USA; 4 South Texas Center for Emerging Infectious Diseases, University of Texas at San Antonio, San Antonio, TX 78249, USA

**Keywords:** Protease, malaria, *Plasmodium*, phylogenomics, genomics, target, vaccine, systems biology, remote homology detection.

## Abstract

Malaria continues to be one of the most devastating global health problems due to the high morbidity and mortality it causes in endemic regions. The search for new antimalarial targets is of high priority because of the increasing prevalence of drug resistance in malaria parasites. Malarial proteases constitute a class of promising therapeutic targets as they play important roles in the parasite life cycle and it is possible to design and screen for specific protease inhibitors. In this mini-review, we provide a phylogenomic overview of malarial proteases. An evolutionary perspective on the origin and divergence of these proteases will provide insights into the adaptive mechanisms of parasite growth, development, infection, and pathogenesis.B

## INTRODUCTION

Malaria is one of the most important and persistent global infectious diseases. It is re-emerging as the number one infectious killer, responsible for over one million deaths yearly. The causative agents of malaria are a group of parasites in the *Plasmodium* genus. Five species, *P. falciparum*, *P. vivax*, *P. malariae*, *P. ovale*, and *P. knowlesi* are human pathogens. *P. falciparum* causes the most deadly form of malaria.

The re-emergence of malaria is largely due to the growing prevalence of parasite populations that show resistance to multiple drug treatment. With the advent of high throughput genomic, transcriptomic, proteomic, metabolomic, and pharmacogenomic technologies, enormous efforts have been focused on the identification and characterization of new and effective antimalarial targets [1-7]. These targets are selected based on several common criteria: (1) They are essential for parasite biology. The disruption of these genes or gene products leads to deleterious effects on parasite growth, development, or invasion. For example, cyclin-dependent protein kinases (CDKs) play indispensible roles in cell cycle progression and signal transduction [8-17]; (2) It is feasible to design or screen for effective pharmacophores or candidate inhibitors. For example, two compounds, chalcones and tryptanthrins, were identified by rational drug design, compound screening and molecular modeling as potent and specific inhibitors for the *P. falciparum* CDK7 homolog, Pfmrk [18]; (3) The drugs directed at the selected targets should have no or minimal adverse effects on humans. Some of the potential targets such as 1-deoxy-D-xylulose 5-phosphate (DOXP) reducto-isomerase [19, 20] and apicoplast gyrase [21] are localized to apicoplast, an organelle uniquely present in *Plasmodium* parasites and other parasites in the *Apicomplexa* phylum. These enzymes are crucial for apicoplast metabolism, replication, transcription and translation. Because the apicoplast is of prokaryotic origin, the inhibitors of these targets may have small or no side effects on the host. 

Proteases are a class of promising antimalarial targets. They are digestive enzymes that degrade peptide bonds. They have demonstrated roles in parasite nutrition, development, invasion and egress: (1) a cascade of aspartic proteases plasmepsins [22, 23], cysteine proteases falcipains [24, 25] and metalloproteases [26-28] mediate massive degradation of host hemoglobin to release amino acids for parasite nutrition; (2) serine proteases (subtilases) have been implicated in erythrocyte invasion and parasite exit from the host [29-32]; (3) proteases are active mediators for cell cycle regulation and cell signaling [33-35]. Because the mechanisms of enzymatic action for many classes of proteases are known or can be derived from structural modeling or computer-aided drug design, it is possible to design or screen for protease inhibitors. The inhibitor classes for plasmepsins and falcipains have been investigated and evaluated [36-44].

Proteases, in addition to their potential as drug targets, are a prime example of supergene families with complex evolutionary histories involving gene duplication, domain shuffling, and lateral gene transfer. In this paper, we present a phylogenomic survey of malarial proteases. A better understanding of protease evolution will bring new insights into the genetic basis of adaptive phenotypes such as pathogenesis and virulence.

## PHYLOGENEOMICS FOR THE *IN SILICO* PREDICTION OF PROTEASES IN THE *PLASMODIUM* GENOMES

Phylogenomics is an emerging discipline that combines molecular evolution theory and genomics [45, 46]. One of its direct and most important applications is to make functional predictions for previously uncharacterized proteins. The major hurdle that plagues all genomics-driven efforts in antimalarial target identification is the annotation problem [47]. In *Plasmodium* species, sequence similarity can be low, due to mutation, insertion, deletion, shuffling and recombination events, meaning high-confidence alignments between descendant sequences are not feasible and functional assignments are obscured. Genome annotation using traditional alignment-based algorithms has failed to assign functionality to over 60% of the ORFs in *P. falciparum* [48]. Popular methods for building probabilistic alignment models, such as PSI-BLAST [49], hidden Markov models (HMMs) [50], COMPASS [51] and HHSearch [52] show low accuracy and coverage when sequence similarity falls below 30% [53-55]. 

Only a handful of proteases had been discovered and characterized prior to the completion of genome sequencing for *P. falciparum* [48]. Using a comparative genomic approach, we predicted that a total of 92 protease homologs were present in *P. falciparum* genome, and at least 88 of them were expressed at the mRNA level by microarray and RT-PCR assays [56]. Subsequent data mining on the parasite proteome revealed that 67 of these predicted proteases were expressed at the protein level at least in one stage of the life cycle [57]. Recently we extended our study to other sibling species of malaria parasites, including *P. vivax* [58], which is the most widely distributed human malaria parasite, and three rodent species *P. berghei*, *P. chabaudi*, and *P. yoelii yoelii* [59, 60], which serve as the animal models for human malaria. In addition to traditional BLAST searches, we adopted a novel support vector machine (SVM)-based, supervised machine learning approach to tackle the remote homology problem. The underlying principle for remote homology detection lies in the domain of phylogenomics: these algorithms are designed to capture subtle similarities between the unknown proteins and the annotated proteins based on the evolutionarily conserved characteristics of the genes/proteins. A SVM classifier is a function that separates the training data into two classes and also maximizes the geometric margin between them in a feature space. Unlike most alignment-based algorithms which build models only with positive sequences, SVMs also use negative sequences (proteins outside the protein family) to learn the difference between the two classes. The SVM approach discovered several putative proteases that were not detectable by PSI-BLAST. For example, one putative PPPDE protease (PFI0940c) is a member of a novel family with a papain-like fold. This family was postulated to play a role in deubiquitination and cell cycle regulation [61]. The total number of predicted proteases in *P. falciparum* was increased from 92 to 123. 

The degradome of five malaria parasite species is comprised of 115-137 putative proteases in five distinct catalytic classes (aspartic, cysteine, metallo, serine and threonine) (See Table **2** and Table **3** in [47]), which account for 0.9-2.3% of the open reading frames (ORFs) in the genome. They form 37 protein families based on their evolutionary relationship and structure conservation, according to the MEROPS protease classification system [62], and 29 of these families are commonly shared in five species. These proteases are important players in metabolism, cell cycle regulation, invasion, stress response, transcriptional regulation, signal transduction, and trafficking. A number of these proteases are becoming targets for functional characterization and rational inhibitor design [43, 63-69]. 

## PHYLOGENOMICS FOR FUNCTIONAL CHARACTERIZATION OF MALARIAL PROTEASES

Phylogenomic analysis provides a cost-effective means to examine the evolutionary profiles of genes and gene products for functional prediction or characterization. The procedure often involves homology identification, multiple sequence alignment, phylogenetic reconstruction, inference of function, evolutionary analysis of orthology and paralogy, and identification of lateral gene transfer events [70]. This approach is particularly useful for the studies of protein families and protein superfamilies such as kinases and transporters [71-74]. In the domain of protease researches, phylogenomics has contributed to, for example, the classification and reconstruction of evolutionary diversification of serine proteases in fungi [75], the evolutionary profiling of cystatins, which comprise a superfamily of cysteine protease inhibitors [76], and the development of a statistical framework that was able to detect site-specific functional divergence in the caspase family of cysteine proteases [77]. The annotation of the predicted malarial proteases was essentially based on phylogenomic analysis [56], which revealed an array of novel proteases that could potentially be important for parasite-specific functions. A single copy of calpain was identified in the five surveyed *Plasmodium* genomes. It contains active site residues (C1035-N-1371-H1391 in *P. falciparum*) that are conserved in known or characterized calpains. Although calpains are well-known modulators for signal transduction, differentiation, cell motility, cell cycle regulation and cell-cell communication from bacteria to humans, its physiological role in parasite biology is yet to be defined. Nevertheless, partial knockdown assays indicated that the malarial calpain is crucial for the optimal growth of the parasite and cell cycle progression [78]. Interestingly, phylogenetic analysis revealed that the malarial calpain belongs to a clade of calcium-independent calpains, a lineage restricted to alveolates; the divergence from major human calpains makes it a possible drug target. 

**Fig. (1):**
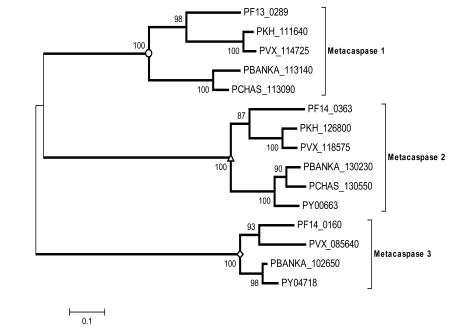
The phylogenetic tree of metacaspases in *Plasmodium*, inferred using the neighbor-joining method based on the amino acid sequences with Poisson corrected distance [111]. Evolutionary analyses were conducted in MEGA5 [112]. The option of complete deletion of gaps was used for tree construction. 1,000 bootstrap replicates were used to infer the reliability of branching points. Bootstrap values of >50% are presented. The scale bar indicates the number of amino acid substitutions per site. The abbreviations for species names are: Pf: *P. falciparum*; PKH: *P. knowlesi*; PVX: *P. vivax*; PBNKA: *P. berghei* ANKA; PCHAS: *P. chabaudi* AS; PY: *P. yoelli yoelii.*

**Fig. (2):**
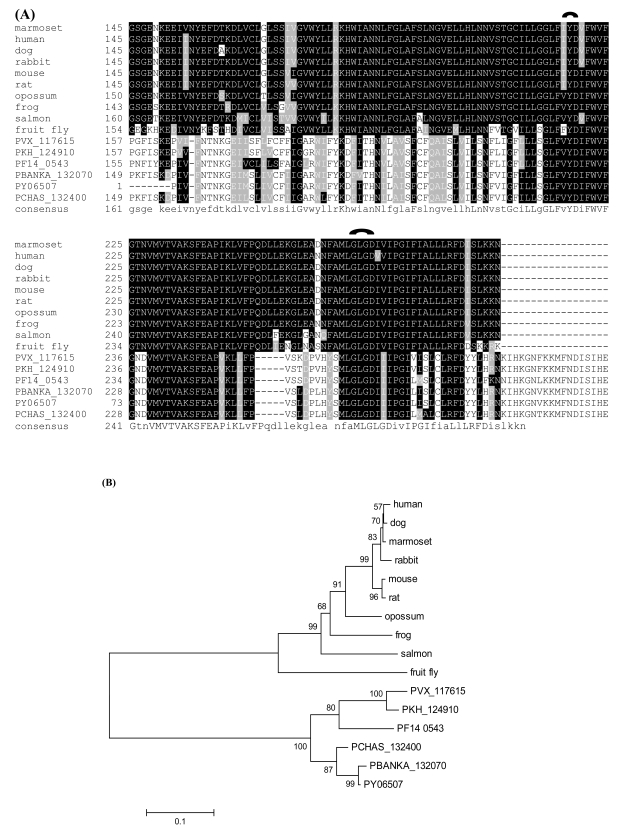
(**A**) The alignment of the catalytic region of the signal peptide peptidases (SPPs) in six *Plasmodium* species and other representative species. The putative active sites Tyr–Asp (YD) and Gly–Leu–Gly–Asp (GLGD) motifs are highlighted. (**B**) The phylogenetic tree of SPPs, inferred using the neighbor-joining method based on the amino acid sequences with Poisson corrected distance.

Multiple copies of metacaspases were identified in *Plasmodium* genomes [34, 47, 79], suggesting the existence of apoptosis or a similar signaling cascade in malaria parasites. Apoptosis-inducer and the administration of antimalarial drug chloroquine were shown to lead to DNA fragmentation and mitochondrial membrane potential disruption, indicative of the onset of programmed cell death in the parasite [80]. Phylogenetic analysis shows that this family may be generated by at least one gene duplication event: metacaspase-3 may represent an ancestral form, while metacaspase-1 and metacaspase-2 are more closely related to each other (Fig. **1**). Metacaspase-1, in particular, contains the typical catalytic domain and the active site residues (histidine and cysteine dyad) that are essential for proteolytic function. In addition, it harbors a caspase recruitment domain (CARD) for apoptosis-related signaling [80]. The discovery of metacaspases has triggered the search for proteins/regulators in the parasite apoptotic network [81], which may represent a major stress-response system for parasites to survive under drug treatment and host immune challenges. Another example of potentially important proteases in malaria parasites is the signal peptide peptidase (SPP). SPP is an active player in regulated intramembrane proteolysis (RIP), which initiates signal transduction *via* processing the transmembrane segments of the substrates. One single copy of SPP is found in *P. falciparum*, *P. vivax*, *P. yoelii yoelii*, and *P. chabaudi*, and two copies are present in *P. berghei.* The putative active sites Tyr–Asp (YD) and Gly–Leu–Gly–Asp (GLGD) motifs which are universally conserved in SPPs are present in all the malarial SPPs (Fig. **2A**). Phylogenetic analysis shows that the malarial SPPs form a distinct clade that is distantly related to the SPPs found in other animals from the fruit fly to the human (Fig. **2B**). Data mining of the protein-protein interaction network revealed that *P. falciparum* SPP (PfSPP) is a highly connected protein with 54 association partners. Fig. (**3**) shows a schematic association map of PfSPP with representative partners with relatively high statistical support. Each association between a pair of proteins has a confidence score (S) ranging from 0.15 to 0.999 that was inferred from the evidence used to establish the association [82], including the interolog comparisons, which is rooted on phylogenomic inference of network associations among evolutionarily related organisms, yeast-2-hybrid (Y2H) assays, Gene Ontology (GO) classification for biological functions, cellular processes and sublocations, structural configuration, co-expression and co-occurrence patterns, and so on [83, 84]. Phylogenomic inference predicted that PfSPP is associated with a putative ER lumen protein retaining receptor (ERD2) [85] and a secretory protein Sec61 [86], both of which are components in the parasite translocation machinery required for the uptake of nutrients and expulsion of wastes: the SPP homolog was found to be co-expressed with the ERD2 and Sec61 homologs in three model organisms: *Arabidopsis thaliana*, *Caenorhabditis elegans*, and *Drosophila melanogaster.* PfAPP is also associated with signal peptidase, translation initiation and elongation factors, splicing factor, peptide chain release factor, and a variety of enzymes, suggesting that it is involved in transport, translation, posttranslational regulation and metabolism. It has recently been considered as a promising drug target since gene disruption assays indicate it is essential for parasite growth and merozoite invasion [87, 88].

## PHYLOGENOMICS FOR ASSESSING THE SUITABILITY OF MALARIAL PROTEASES AS DRUG TARGETS

**Fig. (4):**
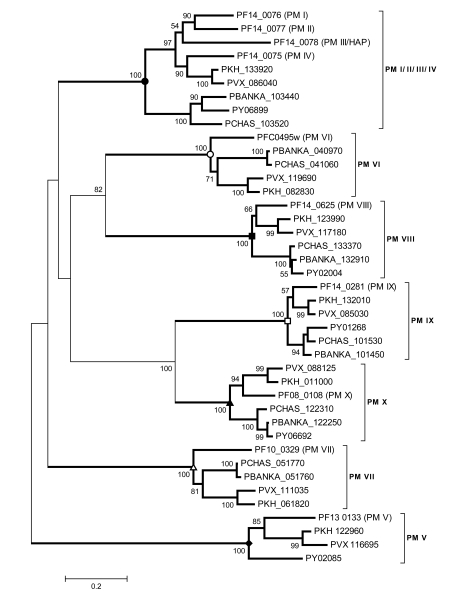
The phylogenetic tree of plasmpepsins, inferred using the neighbor-joining method based on the amino acid sequences with Poisson corrected distance. PM I-IV contain one single exon without any introns. PM VI contains 15 exons, PM VIII contains 13 exons, PM VII and PM IX contain eight exons, and PM V and PM X contain one exon.

Phylogenomic analysis can reveal the complex evolutionary history of malarial proteases; their origin and relatedness to the host help researchers to assess their suitability as potential antimalaria targets. Jean *et al.* [89] and Coombs *et al.* [22] conducted elegant phylogenomic analyses on plasmepsins, a group of aspartic proteases in the pepsin (A1) family. Ten plasmepsins have been identified in *P. falciparum*, namely PM I-X; PM III is also known as histo-aspartic protease (HAP). They are divided into two classes (Fig. **4**): (1) PM I-IV are all intronless with one single exon; They are located in adjacent positions on Chromosome 14, and are likely to be generated by tandem gene duplications. (2) PM V-X form a large clade which may represent an ancestral type of plasmepsins; Introns are present in PM VI, VII, VIII, and IX. The most extreme case is seen in PM VI, which contains 15 exons. An evolutionary model suggested that lateral gene transfer, exon shuffling and intron loss events may lead to the diverse types of plasmepsins in the parasite genome that are required for effective hemoglobin digestion [22, 89]. Inhibition of hemoglobin digestion causes starvation of the parasite and accumulation of intermediates toxic to parasites [23, 90]. Recently, two neutral aminopeptidases, M1 alanyl aminopeptidase (PfM1AAP, MAL13P1.56) and M17 leucine aminopeptidase (PfM17LAP, PF14_0439) have been characterized. Both enzymes are required for the late stage of hemoglobin digestion, which releases free amino acids for parasite nutrition and development inside the human red blood cell. Parasites were not viable *in vivo* and *in vitro* with the treatment of aminopeptidase inhibitors [91]. Because only single copy of PfM17LAP is present in *P. falciparum* genome, its disruption cannot be compensated for by any homolog. The X-ray structure of PfM17LAP has been resolved, opening a promising avenue for rational drug design [92]. 

Gene duplication and lateral gene transfer are implicated in the evolution of other protease families such as subtilases and falcipains. Three copies of subtilases are found in *P. falciparum* genome and 2-5 copies are present in the other *Plasmodium* genomes. Subtilases are required for parasite invasion and egress from the human host [30, 93-95]. Evolutionarily, they are probably acquired *via *lateral gene transfer from a bacteria origin where subtilsins are commonly found. No statistically significant subtilase homologs are found in the humans, which is a desirable feature for drug targets. Similarly, falcipains are crucial for parasite biology; they may have dual roles in both hemoglobin digestion and host cell egress [30, 96-98]. They are evolutionarily closely related to the papains found in viruses and fungi. Lineage-specific expansion is evident in the evolution of rhomboid proteases (the S54 serine protease family) in *Plasmodium*: eight copies are present in *P. falciparum*, and 5-8 copies are present in the other species. They are the central players in regulated intramembrane proteolysis (RIP) and have been implicated in parasite development, invasion, cell signaling and pathogenesis [35, 99-101]. Different rhomboid proteases may have specific substrates preferences; their potential substrates include various adhesins and surface antigens [33]. Phylogenetic analysis revealed that they are closely related to the rhomboid homologs present in other apicomplexan parasites including *Toxoplasma gondii*, *Eimeria tenella*, *Cryptosporidium spp.* and *Theileria spp* [102]. There is only weak sequence similarity between the malarial rhomboids and a mitochondrial rhomboid protease, PARL, in the human genome. Targeting rhomboids and their associated signaling pathways therefore may be a novel therapeutic strategy. Other proteases with potentially important functions, such as calpain, metacaspases, and signal peptidase I, are also phylogenetically divergent from the host lineage.

Phylogenomic analysis also revealed a group of putative malarial proteases that are destined to parasite-specific organelles of prokaryotic origin. The top target organelle for drug development is the apicoplast. It is essential for parasite life cycle, as inhibitors for apicoplast metabolism and replication resulted in the death of parasite [103, 104]. Using two independent algorithms, PATS (an artificial neural network algorithm) [105] and PlasmoAP [106], which combines signal peptide prediction [107] and rule-based classification, Ralph *et al.* [104] identified more than 540 genes in *P. falciparum* that are targeted to the apicoplast. Twenty-one of these genes encode putative proteases (Table **1**). Because the apicoplast is derived from an ancient endosymbiosis in which the eukaryotic ancestor engulfed a red alga with a solitary chloroplast, its disruption does not cause significant interference with the host functions. The cyanobacterial heritage of the apicoplast enhances the potential of these apicoplast-targeted proteases as drug targets. Notably, five putative proteases from the ClpP endopeptidase family (S14) are predicted to be localized to the apicoplast (Table **1**). They are the central players in the parasite heat shock response system, a key system for parasite adaptation to host environment, which involves a transmission from the *Anopheles* *gambiae* mosquito (~25°C) to the human host (~37°C), and the host's recurrent fever caused by infection. Fig. (**5**) shows a protein-protein association map of a putative Clp protease, PF11_0175. It is associated with various heat shock proteins (HSPs) including HslV (PFL1465c), an ATP-dependent threonine protease, Hsp60 (PF10_0153), Hsp70 homologs (PF11_0351, PF08_0054, PFI0875w, and MAL7P1.228), Hsp 90 (PF07_0029), Hsp40 (PFB0595w), a putative small Hsp (PF13_0021), a chaperonin cpn10 (PFL0740c), a putative Hsp70/Hsp90 organizing protein (PF14_0324), and a co-chaperone GrpE(PF11_0258). Inhibition assays showed that the Clp proteases and the chaperone HSPs are essential for parasite growth and development [108, 109]. It is possible to design inhibitors targeting specifically for malarial Clp proteases as the selective inhibitors for the ClpP protease complex has been developed in the bacteria system in *Staphylococcus aureus* [110].

## CONCLUSIONS

The phylogenomic approach has played an important role in the identification and *in silico* characterization of proteases in malaria parasites, providing a promising and largely uncharacterized set of targets for wet lab functional characterization and drug design. An evolutionary perspective on the origin and divergence of these proteases provides insights into the adaptive mechanisms of parasite growth, development, infection, and pathogenesis. 

## Figures and Tables

**Fig. (3):**
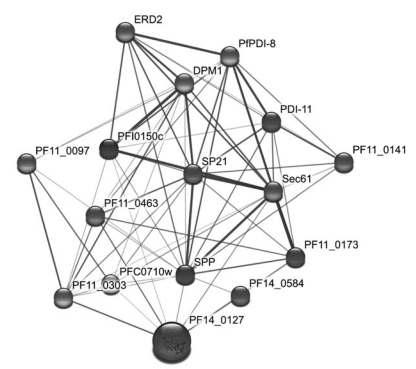
The protein-protein association map of *Plasmodium falciparum* SPP. The association partners were predicted by STRING [84]. This set of associations can be visualized in Cytoscape [113] and converted to an undirected weighted graph. Confidence scores for the interactions among the nodes (S values from STRING) were divided into three groups - low (0.150-0.399), medium (0.400-0.700) and high (0.701-0.999); the groups are represented by thin, medium and heavy lines, respectively.

**Fig. (5):**
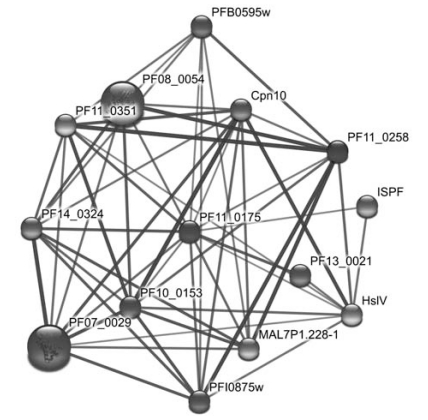
The protein-protein association map of PF11_0175, a putative Clp protease in *P. falciparum.*

**Table 1:** Putative Proteases that are Predicted to Localize to the Apicoplast in P. *falciparum*

Catalytic Type	Protease Family	*P. falciparum *protease ID	Annotation
Aspartic	A1 (pepsin family)	PF13_0133	Plasmepsin V
		PF14_0625	Plasmepsin VIII
	A22 (presenilin family)	PF14_0543	signal peptide peptidase
Cysteine	C1 (papain family)	PFD0230c	dipeptidyl peptidase 3
Metallo	M1 (aminopeptidase N family)	MAL13P1.56	M1-family alanyl aminopeptidase AMPN
	M16 (pitrilysin family)	PF14_0382	Pitrilysin
	M17 (leucyl aminopeptidase family)	PF14_0439	M17 leucyl aminopeptidase
	M24 (methionyl aminopeptidase 1)	MAL8P1.140	putative methionine aminopeptidase 1c
		PF14_0517	aminopeptidase P
	M41 (FtsH endopeptidase family)	PF14_0616	putative ATP-dependent protease la
Serine	S8 (subtilisin family)	PFE0355c	putative subtilisin-like protease 3
	S14 (ClpP endopeptidase family)	PFC0310c	ATP-dependent Clp protease proteolytic subunit
		PF14_0348	ATP-dependent Clp protease proteolytic subunit
		PF08_0063	putative ClpB
		PF14_0063	putative ATP-dependent Clp protease
		PF11_0175	Clp protease, heat shock protein 101
	S33 (prolyl aminopeptidase)	PFC0065C	putative alpha/beta hydrolase
		PF14_0015	putative aminopeptidase
	S54 (Rhomboid family)	MAL8P1.16	rhomboid protease ROM3
		PF13_0312	rhomboid protease ROM7
Unknown	U48 (prenyl protease 2 family)	PFI0660c	putative protease

## ABBREVIATIONS

CARD: = Caspase recruitment domain
CDK: = Cyclin-dependent protein kinase
DOXP: = 1-Deoxy-D-xylulose 5-phosphate
ER: = Endoplasmic reticulum
GO: = Gene Ontology
HAP: = Histo-aspartic protease
HSP: = Heat shock protein
HMM: = Hidden Markov models
ORF: = Open reading frame
RIP: = Regulated intramembrane proteolysis
SPP: = Signal peptide peptidase
SVM: = Support vector machine
Y2H: = Yeast 2-hybrid
